# Sitosterolemia Due to a New Combination of *ABCG8* Variants Presenting as Hemolytic Anemia and Macrothrombocytopenia

**DOI:** 10.1210/jcemcr/luaf234

**Published:** 2025-10-09

**Authors:** Anum, Natalie R Bavli, Zahid Ahmad

**Affiliations:** Division of Endocrinology, Department of Medicine, University of Texas Southwestern Medical Center, Dallas, TX 75390-8537, USA; Division of Hematology and Oncology, Department of Medicine, University of Texas Southwestern Medical Center, Dallas, TX 75390-8537, USA; Division of Endocrinology, Department of Medicine, University of Texas Southwestern Medical Center, Dallas, TX 75390-8537, USA

**Keywords:** sitosterolemia, *ABCG8*, macrothrombocytopenia, stomatocytosis, hemolytic anemia

## Abstract

Sitosterolemia is a rare, autosomal recessive lipid disorder caused by mutations in adenosine triphosphate–binding cassette subfamily G member 5 or 8 (*ABCG5/8*), leading to excessive absorption and systemic accumulation of plant sterols. Patients typically present with xanthelasma, tendon xanthomas, premature atherosclerosis, and hematologic abnormalities. We report a case of sitosterolemia in a man presenting with hemolytic anemia, macrothrombocytopenia, and hypercholesterolemia. Genetic testing revealed a novel combination of *ABCG8* variants, expanding the known spectrum of pathogenic mutations associated with this condition. The patient was treated with statin, ezetimibe, and low plant sterol diet, and after 9 months his plasma sterol levels improved.

## Introduction

Sterols are lipophilic substances formed from squalene conversion into stigmasterol, sitosterol, campesterol, and ergosterol. In a typical Western diet, plant sterols (phytosterols) are ingested through nuts, seeds, legumes, and vegetable oils. Plant sterols and cholesterol are absorbed by the Niemann-Pick C1-like 1 (NPC1L1) protein in enterocytes. Under normal physiological conditions, nearly all absorbed phytosterols are excreted in bile via adenosine triphosphate–binding cassette subfamily G member 5 and 8 (*ABCG5*/8) transporters, with less than 5% retained [1].

Sitosterolemia, first described in 1974 [2], is caused by biallelic loss-of-function variants in either *ABCG5* or *ABCG8*, leading to increased intestinal absorption and systemic accumulation of plant sterols [3]. Patients classically present with tendinous and tuberous xanthomas and premature coronary atherosclerosis [4, 5], but may also develop hemolysis, splenomegaly, platelet abnormalities, and arthritis [6]. Diagnosis is confirmed by measuring plasma plant sterol levels using gas chromatography–mass spectrometry or liquid chromatography–mass spectrometry, along with genetic sequencing of *ABCG5* and *ABCG8* [3].

Management focuses on reducing dietary intake of cholesterol and plant sterols, although achieving a sterol-free diet is challenging. Bile acid sequestrants can reduce plasma sterol levels by disrupting enterohepatic circulation. Ezetimibe, which inhibits NPC1L1-mediated absorption, is the preferred pharmacologic agent for sitosterolemia and effectively lowers plant sterol levels [7].

Around 50 different mutations have been reported in the literature in the *ABCG8* gene associated with sitosterolemia. This case reports biallelic missense variants including the co-occurrence of 2 missense variants on the same allele.

## Case Presentation

A 61-year-old man with a history of hypothyroidism, hypercholesterolemia, obesity, and metabolic dysfunction–associated steatotic liver disease was referred to the hematology clinic at University of Texas Southwestern for the evaluation of hemolytic anemia and thrombocytopenia. He had previously been empirically treated with corticosteroids by an outside hematologist, without clinical improvement in his platelet count. Hypercholesterolemia was diagnosed in early adulthood, and he had been taking rosuvastatin 5 mg daily for several years.

Family history was significant for both parents taking statins (presumably due to hyperlipidemia) and a maternal grandmother with hyperlipidemia. No relative was diagnosed with sitosterolemia. His mother had several strokes, with the first one in her 60s. Both paternal grandparents had a history of stroke and both maternal grandparents had coronary artery disease. One sister had a stroke. He has 2 kids, and both were healthy.

## Diagnostic Assessment

**Figure 1.**
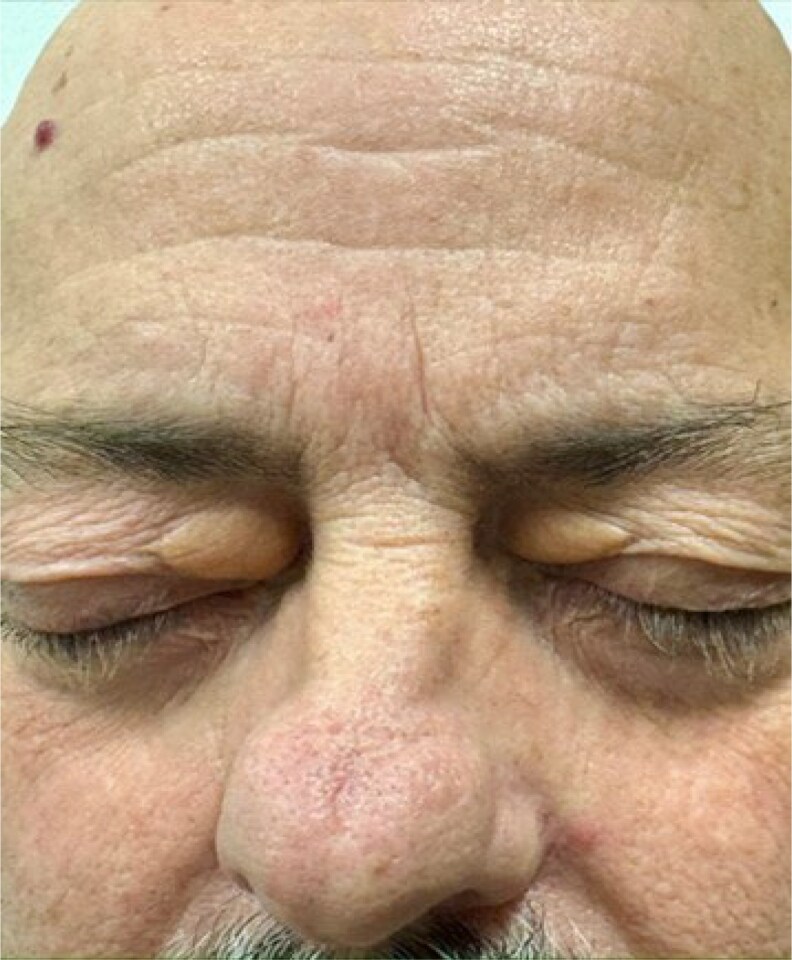
Bilateral xanthelasma.

**Figure 2.**
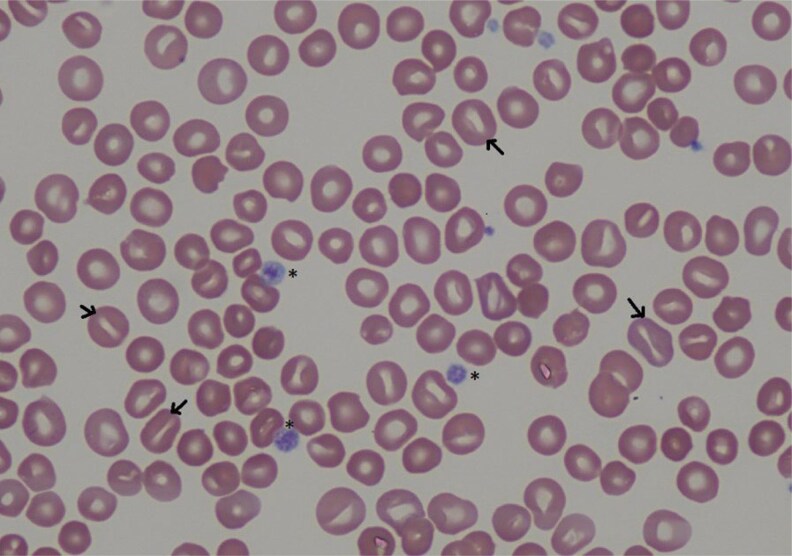
Asterisks indicate large platelets; black arrows indicate stomatocytes.

On examination, the patient had splenomegaly and bilateral xanthelasma (Fig. 1). Laboratory testing revealed a low-density lipoprotein of 126 mg/dL (SI 3.27 mmol/L), anemia (hemoglobin 11.5 g/dL [SI: 115 g/L]), thrombocytopenia (platelets 75 × 10^3^/µL [SI: 75 × 10^9^/L]), elevated lactate dehydrogenase (308 U/L [SI: 5.13 ukat/L]), and undetectable haptoglobin (Table 1). A peripheral blood smear revealed marked stomatocytosis and macrothrombocytopenia (Fig. 2). Given the constellation of hemolytic anemia, macrothrombocytopenia with stomatocytosis, and xanthelasma, sitosterolemia was suspected.

**Table 1.** Laboratory parameters of patient on initial and follow-up visits

Laboratory parameters	Baseline	At 3 mo follow-up	At 6 mo follow-up	At 9 mo follow-up	Normal values
Hemoglobin	11.5 g/dL (115 g/L)	12.4 g/dL (124 g/L)	12.5 g/dL (125 g/L)	13.4 g/dL (134 g/L)	12.4-17.3 g/dL (124-173 g/L)
Hematocrit	35.1% (0.35)	40% (0.40)	40.8% (0.40)	43.4% (0.43)	37-50% (0.37-0.5)
RBC	3.66 million/mm (3.66 × 10^12^/L)	3.98 million/mm^3^ (3.98 × 10^12^/L)	4.08 million/mm^3^ (4.08 × 10^12^/L)	4.59 million/mm^3^ (4.59 × 10^12^/L)	4-5.8 million/mm^3^ (4.00-5.80 × 10^12^/L)
Platelets	75 × 10^3^/µL (75 × 10^9^/L)	70 × 10^3^/µL (70 × 10^9^/L)	71 × 10^3^/µL (71 × 10^9^/L)	85 × 10^3^/µL (85 × 10^9^/L)	150-450 × 10^3^/µL (150-450 × 10^9^/L)
Cholesterol	219 mg/dL (5.7 mmol/L)	151 mg/dL (3.9 mmol/L)	186 mg/dL (4.8 mmol/L)	176 mg/dL (4.6 mmol/L)	<200 mg/dL (<5.18 mmol/L)
LDL-C	126.4 mg/dL (3.27 mmol/L)	81.4 mg/dL (2.11 mmol/L)	107.4 mg/dL (2.78 mmol/L)	97.6 mg/dL (2.52 mmol/L)	≤100 mg/dL (<2.59 mmol/L)
HDL	60 mg/dL (1.55 mmol/L)	48 mg/dL (1.24 mmol/L)	57 mg/dL (1.47 mmol/L)	54 mg/dL (1.4 mmol/L)	>39 mg/dL (>1.01 mmol/L)
Triglycerides	163 mg/dL (1.8 mmol/L)	108 mg/dL (1.2 mmol/L)	108 mg/dL (1.2 mmol/L)	122 mg/dL (1.4 mmol/L)	<150 mg/dL (<1.70 mmol/L)
Non-HDL	159 mg/dL (4.12 mmol/L)	103 mg/dL (2.67 mmol/L)	129 mg/dL (3.34 mmol/L)	122 mg/dL (3.16 mmol/L)	95-160 mg/dL (2.46-4.14 mmol/L)
7-Dehydrocholesterol	0.3 mg/L (0.006 mmol/L)	0.3 mg/L (0.006 mmol/L)	0.2 mg/L (0.004 mmol/L)	0.3 mg/L (0.006 mmol/L)	≤2.0 mg/L (0.04 mmol/L)
8-Dehydrocholesterol	0.2 mg/L (0.004 mmol/L)	0.1 mg/L (0.002 mmol/L)	0.2 mg/L (0.004 mmol/L)	0.3 mg/L (0.006 mmol/L)	≤0.3 mg/L (≤ 0.006 mmol/L)
8(9)-Cholestenol	<1.0 mg/L (<0.02 mmol/L)	<1.0 mg/L (<0.02 mmol/L)	<1.0 mg/L (<0.02 mmol/L)	<1.0 mg/L (<0.02 mmol/L)	≤5.0 mg/L (≤0.10 mmol/L)
Campesterol	189.6 mg/L (4.12 mmol/L)	152.5 mg/L (3.31 mmol/L)	88.8 mg/L (1.93 mmol/L)	97.5 mg/L2.11 mmol/L)	≤8.0 mg/L (≤ 0.17 mmol/L)
Cholestanol	13.6 mg/L (0.29 mmol/L)	12.7 mg/L (0.27 mmol/L)	11.9 mg/L (0.25 mmol/L)	16.7 mg/L (0.36 mmol/L)	≤6.0 mg/L (≤ 0.13 mmol/L)
Desmosterol	0.8 mg/L (0.017 mmol/L)	0.6 mg/L (0.013 mmol/L)	0.5 mg/L (0.01 mmol/L)	0.6 mg/L (0.013 mmol/L)	≤2.5 mg/L (≤ 0.05 mmol/L)
DiHydro T-MAS	0.7 mg/L (0.015 mmol/L)	0.1 mg/L (0.002 mmol/L)	0.3 mg/L (0.006 mmol/L)	0.2 mg/L (0.004 mmol/L)	≤0.3 mg/L (≤ 0.006 mmol/L)
Lathosterol	1.7 mg/L (0.036 mmol/L)	1.5 mg/L (0.032 mmol/L)	0.6 mg/L (0.013 mmol/L)	1.0 mg/L (0.02 mmol/L)	≤6.0 mg/L (≤0.13 mmol/L)
Sitosterol	374.4 mg/L (8.13 mmol/L)	328.3 mg/L (7.13 mmol/L)	203.4 mg/L (4.42 mmol/L)	199.8 mg/L (4.34 mmol/L)	≤15.0 mg/L (≤0.32 mmol/L)
Squalene	0.5 mg/L (0.01 mmol/L)	0.2 mg/L (0.004 mmol/L)	0.2 mg/L (0.004 mmol/L)	0.2 mg/L (0.004 mmol/L)	≤1.0 mg/L (≤ 0.02 mmol/L)
Stigmasterol	20.3 mg/L (0.44 mmol/L)	12.6 mg/L (0.27 mmol/L)	8.5 mg/L (0.18 mmol/L)	10.2 mg/L (0.22 mmol/L)	≤0.5 mg/L (≤ 0.01 mmol/L)

Abbreviations: HDL, high-density lipoprotein; LDL, low-density lipoprotein; LDL-C, low-density lipoprotein cholesterol; RBC, red blood cells.

Serum plant sterol levels were markedly elevated (see Table 1). Genetic testing identified multiple heterozygous variants in *ABCG8*: a likely pathogenic *c.250_280dup* variant on 1 allele, and 2 variants (*c.1721G*  *>*  *A* [p. Gly574Glu] and *c.1723G*  *>*  *C* [p. Gly575Arg]) on the other, resulting in a novel deletion-insertion of 2 amino acids. Cascade genetic testing revealed that his asymptomatic sister carried the *c.250_280dup variant*; her plasma sterol levels were normal.

## Treatment

The patient was subsequently referred to the UT Southwestern Lipid Metabolism Clinic, where he was started on ezetimibe 10 mg daily in addition to the rosuvastatin 5 mg daily. A nutritionist guided him in transitioning from a heart-healthy diet to a low-plant-sterol diet.

## Outcome and Follow-up

At 3-, 6-, and 9-month follow-ups, his lipid profile and hemoglobin had improved, while platelet counts remained unchanged. The markers of sterol absorption also improved at 9 months (see Table 1).

## Discussion

The genetic findings in our patient expand the known spectrum of *ABCG8* mutations. The *c.250_280dup* variant results in the duplication of 31 nucleotide at positions c.250 through c.280 of the *ABCG8* gene. It has not been previously reported in sitosterolemia. While the *c.1721G*  *>*  *A* (p. Gly574Glu) and *c.1723G*  *>*  *C* (p. Gly575Arg) missense variants have been individually described [8], their co-occurrence on the same allele represents a novel compound variant. Most reported cases of sitosterolemia in White individuals involve the *ABCG8* mutations, and in prior cases, these variants have typically occurred on different exons [9]. In contrast, our patient's missense variants occur on the same exon, further supporting the pathogenicity of this unique combination.

This case also underscores a rare but often-overlooked cause of hemolytic anemia and thrombocytopenia. Sitosterolemia is frequently misdiagnosed, leading to inappropriate treatments such as corticosteroids or splenectomy. Hematologic abnormalities are among the most common noncardiovascular manifestations of sitosterolemia [10].

In vitro, studies have demonstrated increased osmotic fragility and morphologic distortion of erythrocytes exposed to sitosterol, suggesting plant-sterol incorporation into red cell membranes as a driver for hemolysis [11]. Additionally, 2 mice models of sitosterolemia have shown that plant-sterol accumulation contributes to macrothrombocytopenia [12, 13]. Therefore, the presence of stomatocytosis and macrothrombocytopenia in the setting of dyslipidemia should prompt consideration of sitosterolemia.

A pilot interventional trial suggested that ezetimibe increases the platelet counts and reduces mean platelet volume [14]. However, after 6 months of treatment, our patient's platelet count remained unchanged. Given the variable response of splenomegaly to ezetimibe, with some reports indicating that resolutions may take more than a year, longer follow-up may be necessary [15]. Additionally, our patient experienced only a modest (<50%) decline in plasma sterol levels due to early follow-up, suggesting that clinical response may lag biochemical improvement [16].

## Learning Points

- This case highlights a novel combination of *ABCG8* variants associated with sitosterolemia.
- The identification of previously unreported *ABCG8* mutations broadens the known genetic spectrum of sitosterolemia.
- Clinicians should maintain a high index of suspicion in patients with unexplained hemolytic anemia and macrothrombocytopenia in the setting of lipid abnormalities.

## Contributors

All authors made individual contributions to authorship. N.B. and Z.A. were involved in the diagnosis and management of the patient and reviewed the manuscript. A. reviewed the medical records and collected and organized the data. All authors reviewed and approved the final draft.

## Data Availability

Data sharing is not applicable to this article as no datasets were generated or analyzed during the current study.

## Abbreviations

NPC1L1: Niemann-Pick C1-like 1
